# Supplementary material for: Sleep disorder and apnea events detection framework with high performance using two-tier learning model design
Source: PeerJ Comput Sci. 2023 Sep 29;9:e1554. doi: 10.7717/peerj-cs.1554 (PMC10557519; doi:10.7717/peerj-cs.1554)
Supplement: Supplemental Information 1 [file peerj-cs-09-1554-s001.pdf]

DATA TABLE FOR EACH PATIENT (BEFORE AND AFTER UNDER-SAMPLING)

	Before Undersampling Data Size				After Undersampling Data Size			
PatientID	Apnea	Hypopnea	Normal	Total	Apnea	Hypopnea	Normal	Total
1	5600	2000	4462400	4470000	5600	2000	2000	9600
2	2000	6000	4492000	4500000	2000	6000	2000	10000
3	3400	4000	4630600	4638000	3400	4000	3400	10800
4	3000	6000	4763400	4772400	3000	6000	3000	12000
5	5800	4000	4762600	4772400	5800	4000	4000	13800
6	4800	6000	4477200	4488000	4800	6000	4800	15600
7	14000	2000	4586000	4602000	14000	2000	2000	18000
8	10000	4000	4702000	4716000	10000	4000	4000	18000
9	14200	2000	4747800	4764000	14200	2000	2000	18200
10	3000	16000	4625000	4644000	3000	16000	3000	22000
11	7200	8000	4694800	4710000	7200	8000	7200	22400
12	2000	20000	4322000	4344000	2000	20000	2000	24000
13	4000	18000	4478000	4500000	4000	18000	4000	26000
14	2600	22000	4739400	4764000	2600	22000	2600	27200
15	2000	24000	4746400	4772400	2000	24000	2000	28000
16	7600	14000	4750800	4772400	7600	14000	7600	29200
17	26000	2000	4340000	4368000	26000	2000	2000	30000
18	26000	4000	4530000	4560000	26000	4000	4000	34000
19	8000	18000	4636000	4662000	8000	18000	8000	34000
20	10600	14000	4457400	4482000	10600	14000	10600	35200
21	19400	8000	4544600	4572000	19400	8000	8000	35400
22	24000	6000	4722000	4752000	24000	6000	6000	36000
23	16600	12000	4743800	4772400	16600	12000	12000	40600
24	13600	20000	4394400	4428000	13600	20000	13600	47200
25	14400	20000	4738000	4772400	14400	20000	14400	48800
26	32400	10000	4505600	4548000	32400	10000	10000	52400
27	16800	30000	4627200	4674000	16800	30000	16800	63600
28	14000	36000	4402000	4452000	14000	36000	14000	64000
29	21400	22000	4474600	4518000	21400	22000	21400	64800
30	10200	44400	4553400	4608000	10200	44400	10200	64800
31	8000	52000	4488000	4548000	8000	52000	8000	68000
32	14200	42000	4287800	4344000	14200	42000	14200	70400
33	8800	54000	4455200	4518000	8800	54000	8800	71600
34	28000	22000	4426000	4476000	28000	22000	22000	72000
35	11600	52000	4532400	4596000	11600	52000	11600	75200
36	21200	38000	4500800	4560000	21200	38000	21200	80400
37	21200	44000	4518800	4584000	21200	44000	21200	86400
38	4800	78000	4285200	4368000	4800	78000	4800	87600
39	3200	90000	4679200	4772400	3200	90000	3200	96400
40	21400	54000	4250600	4326000	21400	54000	21400	96800
41	10400	80000	4553600	4644000	10400	80000	10400	100800
42	15200	82000	4276800	4374000	15200	82000	15200	112400
43	31600	54800	4455600	4542000	31600	54800	31600	118000

DATA TABLE FOR EACH PATIENT (BEFORE AND AFTER UNDER-SAMPLING)

44	9800	114000	4250200	4374000	9800	114000	9800	133600
45	18400	100000	4654000	4772400	18400	100000	18400	136800
46	38200	62000	4387800	4488000	38200	62000	38200	138400
47	43400	84000	4588600	4716000	43400	84000	43400	170800
48	20000	132000	4396000	4548000	20000	132000	20000	172000
49	37000	106000	4489000	4632000	37000	106000	37000	180000
50	50000	98000	4526000	4674000	50000	98000	50000	198000
Total Data	761000	1843200	2,27E+08	2,29E+08	761000	1843200	617000	3221200
Average	15220	36864	4533020	4585104	15220	36864	12340	64424
