# Supplementary material for: Sleep disorder and apnea events detection framework with high performance using two-tier learning model design
Source: PeerJ Comput Sci. 2023 Sep 29;9:e1554. doi: 10.7717/peerj-cs.1554 (PMC10557519; doi:10.7717/peerj-cs.1554)
Supplement: Supplemental Information 2 [file peerj-cs-09-1554-s002.pdf]

Table with All Results

	DNN				GRU				RNN				LSTM				Proposed Model			
Patient ID	Accuracy	Precision	Recall	Fscore	Accuracy	Precision	Recall	Fscore	Accuracy	Precision	Recall	Fscore	Accuracy	Precision	Recall	Fscore	Accuracy	Precision	Recall	Fscore
1	0,9427	0,9425	0,9427	0,9426	0,9531	0,9533	0,9531	0,9518	0,9219	0,9206	0,9219	0,9198	0,9597	0,9596	0,9597	0,9589	0,9688	0,9686	0,9688	0,9684
2	0,9603	0,9600	0,9603	0,9599	0,9493	0,9493	0,9493	0,9478	0,9293	0,9279	0,9293	0,9282	0,9517	0,9523	0,9517	0,9498	0,9723	0,9725	0,9723	0,9718
3	0,9772	0,9771	0,9772	0,9771	0,9815	0,9818	0,9815	0,9814	0,9838	0,9844	0,9838	0,9836	0,9787	0,9792	0,9787	0,9786	0,9846	0,9847	0,9846	0,9845
4	0,9614	0,9616	0,9614	0,9610	0,9528	0,9526	0,9528	0,9520	0,9339	0,9331	0,9339	0,9332	0,9525	0,9522	0,9525	0,9522	0,9719	0,9718	0,9719	0,9718
5	0,9783	0,9783	0,9783	0,9782	0,9804	0,9807	0,9804	0,9803	0,9792	0,9794	0,9792	0,9791	0,9862	0,9864	0,9862	0,9862	0,9899	0,9899	0,9899	0,9898
6	0,9380	0,9384	0,9380	0,9381	0,9393	0,9390	0,9393	0,9391	0,9184	0,9183	0,9184	0,9175	0,9427	0,9426	0,9427	0,9426	0,9558	0,9557	0,9558	0,9558
7	0,9804	0,9803	0,9804	0,9797	0,9767	0,9763	0,9767	0,9759	0,9696	0,9691	0,9696	0,9682	0,9800	0,9799	0,9800	0,9793	0,9848	0,9851	0,9848	0,9843
8	0,9600	0,9599	0,9600	0,9594	0,9530	0,9525	0,9530	0,9525	0,9554	0,9550	0,9554	0,9551	0,9656	0,9653	0,9656	0,9651	0,9720	0,9720	0,9720	0,9717
9	0,9742	0,9740	0,9742	0,9735	0,9637	0,9632	0,9637	0,9633	0,9678	0,9673	0,9678	0,9672	0,9659	0,9653	0,9659	0,9653	0,9800	0,9798	0,9800	0,9797
10	0,9658	0,9652	0,9658	0,9651	0,9468	0,9460	0,9468	0,9463	0,9580	0,9579	0,9580	0,9558	0,9567	0,9558	0,9567	0,9549	0,9673	0,9669	0,9673	0,9662
11	0,9516	0,9529	0,9516	0,9509	0,9638	0,9638	0,9638	0,9637	0,9549	0,9553	0,9549	0,9544	0,9606	0,9605	0,9606	0,9605	0,9744	0,9747	0,9744	0,9742
12	0,9708	0,9696	0,9708	0,9692	0,9629	0,9608	0,9629	0,9611	0,9672	0,9656	0,9672	0,9659	0,9674	0,9658	0,9674	0,9659	0,9775	0,9769	0,9775	0,9764
13	0,9847	0,9848	0,9847	0,9845	0,9779	0,9778	0,9779	0,9778	0,9695	0,9692	0,9695	0,9689	0,9828	0,9828	0,9828	0,9826	0,9887	0,9887	0,9887	0,9886
14	0,9788	0,9783	0,9788	0,9784	0,9710	0,9702	0,9710	0,9704	0,9667	0,9656	0,9667	0,9657	0,9739	0,9735	0,9739	0,9728	0,9820	0,9817	0,9820	0,9815
15	0,9636	0,9637	0,9636	0,9636	0,9633	0,9609	0,9633	0,9613	0,9608	0,9586	0,9608	0,9561	0,9454	0,9418	0,9454	0,9389	0,9718	0,9705	0,9718	0,9700
16	0,9255	0,9256	0,9255	0,9248	0,8973	0,8967	0,8973	0,8966	0,8775	0,8774	0,8775	0,8769	0,9129	0,9129	0,9129	0,9122	0,9379	0,9377	0,9379	0,9375
17	0,9776	0,9773	0,9776	0,9769	0,9728	0,9723	0,9728	0,9723	0,9629	0,9617	0,9629	0,9618	0,9728	0,9723	0,9728	0,9721	0,9808	0,9806	0,9808	0,9802
18	0,9668	0,9671	0,9668	0,9665	0,9648	0,9648	0,9648	0,9643	0,9596	0,9594	0,9596	0,9591	0,9653	0,9651	0,9653	0,9650	0,9767	0,9768	0,9767	0,9764
19	0,9808	0,9805	0,9808	0,9802	0,9754	0,9748	0,9754	0,9748	0,9661	0,9651	0,9661	0,9654	0,9762	0,9757	0,9762	0,9755	0,9838	0,9836	0,9838	0,9834
20	0,9504	0,9502	0,9504	0,9502	0,9498	0,9497	0,9498	0,9497	0,9339	0,9336	0,9339	0,9333	0,9574	0,9574	0,9574	0,9571	0,9680	0,9679	0,9680	0,9679
21	0,9819	0,9819	0,9819	0,9818	0,9706	0,9704	0,9706	0,9704	0,9649	0,9648	0,9649	0,9644	0,9770	0,9770	0,9770	0,9768	0,9846	0,9846	0,9846	0,9845
22	0,9486	0,9479	0,9486	0,9472	0,9489	0,9482	0,9489	0,9473	0,9264	0,9247	0,9264	0,9230	0,9468	0,9458	0,9468	0,9460	0,9589	0,9585	0,9589	0,9578
23	0,9716	0,9715	0,9716	0,9715	0,9539	0,9538	0,9539	0,9536	0,9300	0,9297	0,9300	0,9298	0,9566	0,9564	0,9566	0,9563	0,9745	0,9745	0,9745	0,9745
24	0,9335	0,9338	0,9335	0,9330	0,9028	0,9026	0,9028	0,9020	0,8497	0,8502	0,8497	0,8494	0,9133	0,9130	0,9133	0,9127	0,9436	0,9435	0,9436	0,9434
25	0,9474	0,9488	0,9474	0,9470	0,9141	0,9143	0,9141	0,9137	0,9033	0,9043	0,9033	0,9031	0,9284	0,9295	0,9284	0,9281	0,9496	0,9503	0,9496	0,9493
26	0,9695	0,9693	0,9695	0,9690	0,9572	0,9567	0,9572	0,9566	0,9527	0,9521	0,9527	0,9520	0,9622	0,9619	0,9622	0,9617	0,9746	0,9745	0,9746	0,9742
27	0,9626	0,9628	0,9626	0,9624	0,9377	0,9378	0,9377	0,9374	0,9297	0,9304	0,9297	0,9291	0,9439	0,9441	0,9439	0,9435	0,9654	0,9658	0,9654	0,9652
28	0,9299	0,9305	0,9299	0,9287	0,9030	0,9024	0,9030	0,9022	0,8699	0,8697	0,8699	0,8681	0,9149	0,9146	0,9149	0,9142	0,9403	0,9401	0,9403	0,9396
29	0,9230	0,9236	0,9230	0,9232	0,9164	0,9168	0,9164	0,9162	0,8960	0,8958	0,8960	0,8955	0,9179	0,9180	0,9179	0,9175	0,9363	0,9364	0,9363	0,9363
30	0,9550	0,9544	0,9550	0,9545	0,9367	0,9354	0,9367	0,9357	0,9137	0,9114	0,9137	0,9108	0,9482	0,9473	0,9482	0,9475	0,9668	0,9664	0,9668	0,9663
31	0,9597	0,9593	0,9597	0,9579	0,9480	0,9472	0,9480	0,9456	0,9320	0,9303	0,9320	0,9276	0,9444	0,9424	0,9444	0,9423	0,9627	0,9628	0,9627	0,9609
32	0,8878	0,8868	0,8878	0,8848	0,8762	0,8741	0,8762	0,8740	0,8401	0,8389	0,8401	0,8314	0,8974	0,8958	0,8974	0,8937	0,9130	0,9125	0,9130	0,9107
33	0,9588	0,9580	0,9588	0,9573	0,9453	0,9435	0,9453	0,9436	0,9292	0,9261	0,9292	0,9257	0,9483	0,9468	0,9483	0,9468	0,9646	0,9642	0,9646	0,9634
34	0,9538	0,9537	0,9538	0,9536	0,9454	0,9452	0,9454	0,9452	0,9260	0,9257	0,9260	0,9253	0,9473	0,9471	0,9473	0,9470	0,9678	0,9677	0,9678	0,9677
35	0,9442	0,9448	0,9442	0,9426	0,9093	0,9091	0,9093	0,9076	0,8901	0,8887	0,8901	0,8879	0,9284	0,9271	0,9284	0,9271	0,9456	0,9455	0,9456	0,9443
36	0,9123	0,9120	0,9123	0,9119	0,8813	0,8801	0,8813	0,8803	0,8461	0,8445	0,8461	0,8439	0,8892	0,8884	0,8892	0,8886	0,9185	0,9183	0,9185	0,9184
37	0,9412	0,9414	0,9412	0,9401	0,9212	0,9206	0,9212	0,9203	0,9041	0,9036	0,9041	0,9023	0,9313	0,9311	0,9313	0,9302	0,9470	0,9471	0,9470	0,9463
38	0,9656	0,9625	0,9656	0,9623	0,9546	0,9503	0,9546	0,9453	0,9520	0,9456	0,9520	0,9414	0,9557	0,9498	0,9557	0,9501	0,9656	0,9645	0,9656	0,9598
39	0,9799	0,9785	0,9799	0,9766	0,9789	0,9779	0,9789	0,9751	0,9781	0,9780	0,9781	0,9740	0,9790	0,9779	0,9790	0,9754	0,9809	0,9809	0,9809	0,9773
40	0,9274	0,9292	0,9274	0,9247	0,9092	0,9082	0,9092	0,9072	0,8824	0,8809	0,8824	0,8791	0,9150	0,9140	0,9150	0,9131	0,9424	0,9429	0,9424	0,9408
41	0,9240	0,9215	0,9240	0,9189	0,9270	0,9205	0,9270	0,9196	0,8923	0,8907	0,8923	0,8909	0,9238	0,9221	0,9238	0,9219	0,9643	0,9636	0,9643	0,9638

Table with All Results

	DNN				GRU				RNN				LSTM				Proposed Model			
Patient ID	Accuracy	Precision	Recall	Fscore	Accuracy	Precision	Recall	Fscore	Accuracy	Precision	Recall	Fscore	Accuracy	Precision	Recall	Fscore	Accuracy	Precision	Recall	Fscore
42	0,9380	0,9373	0,9380	0,9358	0,9178	0,9156	0,9178	0,9156	0,8968	0,8932	0,8968	0,8915	0,9243	0,9221	0,9243	0,9223	0,9430	0,9420	0,9430	0,9410
43	0,8992	0,8986	0,8992	0,8984	0,8653	0,8648	0,8653	0,8639	0,8241	0,8261	0,8241	0,8206	0,8779	0,8777	0,8779	0,8759	0,9108	0,9104	0,9108	0,9099
44	0,8889	0,8887	0,8889	0,8875	0,8881	0,8868	0,8881	0,8873	0,8763	0,8741	0,8763	0,8740	0,8965	0,8951	0,8965	0,8955	0,9177	0,9177	0,9177	0,9164
45	0,8958	0,8972	0,8958	0,8940	0,8766	0,8746	0,8766	0,8753	0,8542	0,8553	0,8542	0,8539	0,8739	0,8720	0,8739	0,8721	0,9033	0,9019	0,9033	0,9022
46	0,8988	0,8993	0,8988	0,8970	0,9015	0,9002	0,9015	0,9005	0,8811	0,8802	0,8811	0,8775	0,9072	0,9061	0,9072	0,9055	0,9356	0,9349	0,9356	0,9342
47	0,8948	0,8942	0,8948	0,8939	0,8392	0,8377	0,8392	0,8380	0,7884	0,7871	0,7884	0,7860	0,8457	0,8432	0,8457	0,8440	0,9288	0,9280	0,9288	0,9283
48	0,9064	0,9074	0,9064	0,9057	0,9054	0,9076	0,9054	0,9053	0,8863	0,8857	0,8863	0,8858	0,9126	0,9125	0,9126	0,9117	0,9398	0,9395	0,9398	0,9394
49	0,9045	0,9061	0,9045	0,9050	0,8899	0,8901	0,8899	0,8898	0,8588	0,8592	0,8588	0,8584	0,8929	0,8925	0,8929	0,8925	0,9232	0,9231	0,9232	0,9231
50	0,8932	0,8948	0,8932	0,8929	0,8697	0,8698	0,8697	0,8695	0,8391	0,8406	0,8391	0,8393	0,8662	0,8667	0,8662	0,8656	0,9245	0,9244	0,9245	0,9244
**Average**	**0,9457**	**0,9457**	**0,9457**	**0,9448**	**0,9337**	**0,9330**	**0,9337**	**0,9325**	**0,9170**	**0,9162**	**0,9170**	**0,9151**	**0,9384**	**0,9377**	**0,9384**	**0,9372**	**0,9577**	**0,9575**	**0,9577**	**0,9569**
**Min**	**0,8878**	**0,8868**	**0,8878**	**0,8848**	**0,8392**	**0,8377**	**0,8392**	**0,8380**	**0,7884**	**0,7871**	**0,7884**	**0,7860**	**0,8457**	**0,8432**	**0,8457**	**0,8440**	**0,9033**	**0,9019**	**0,9033**	**0,9022**
**Max**	**0,9847**	**0,9848**	**0,9847**	**0,9845**	**0,9815**	**0,9818**	**0,9815**	**0,9814**	**0,9838**	**0,9844**	**0,9838**	**0,9836**	**0,9862**	**0,9864**	**0,9862**	**0,9862**	**0,9899**	**0,9899**	**0,9899**	**0,9898**
